# How experienced nurses think and act in supporting patient learning: an interview study

**DOI:** 10.1186/s12912-025-03542-7

**Published:** 2025-07-07

**Authors:** Tina Gustavell, Charlotte Silén, Lena-Marie Petersson, Lena Engqvist Boman

**Affiliations:** 1 Department of Neurobiology, Care Sciences and Society, Division of Nursing, Karolinska Institutet, Stockholm, 171 77 Sweden; 2 Department of Upper Abdominal Diseases, Karolinska University Hospital, Theme Cancer, Stockholm, Sweden; 3 Department of Learning, Informatics, Management and Ethics, Karolinska Institutet, Stockholm, Sweden; 4 Department of Medicine, Capio St Görans Hospital, Stockholm, Sweden

**Keywords:** Cancer care, Nursing, Patient learning, Patient participation, Pedagogical knowledge, Pedagogical encounters, Person-centred care

## Abstract

**Background:**

Patients’ knowledge and participation are essential for safe care. Nurses must enhance their pedagogical knowledge and skills to support patients to understand their health and become active partners in care. Pedagogical activities were implemented in a specialist nursing program to support this learning. The aim of this study was to explore experienced nurses’ understanding of the meaning of pedagogical encounters with patients in cancer care, as well as how they think and act during these encounters to support patients’ learning.

**Methods:**

Interpretative qualitative study using individual interviews with nurses (*n* = 8) undergoing specialist training. An interview guide along with the nurses’ written assignments during the specialist training were used to aid the interview. Data were analysed using reflexive thematic analysis.

**Results:**

The nurses’ understanding of the meaning of pedagogical encounters with patients in cancer care could be described by one overarching theme “A holistic approach to support patients learning” and five sub-themes: “Supporting patient learning through pedagogical awareness”, “Creating an atmosphere of trust”, “Forming mutual understanding and participation”, “Using personal characteristics and experiences”, and “Engaging in continuous learning and team collaboration”. The nurses’ overarching goal with the pedagogical encounter was to support patients to understand and cope with their situation. They used strategies like active listening, observing non-verbal cues, and seeking feedback to create a supportive, person-centred learning environment. Theoretical knowledge enhanced their awareness of diverse learning methods and how to tailor nursing interactions to individual needs. Still, they emphasized the importance of personal characteristics and experiences over theoretical knowledge, where self-awareness, empathy, and human insight is vital.

**Conclusion:**

The nurses’ thoughts and actions in their encounters with patients were supported by what they had experienced and studied about learning in the program, their competencies in nursing, and most strongly their own personal qualities and experiences. These are all important sources to support patient learning, but we want to forward the importance of including pedagogical knowledge and practice in health care education. Future research should explore how theoretical knowledge in pedagogy can be better integrated into practice.

**Clinical trial number:**

Not applicable.

## Background

Patients’ understanding of their health condition is fundamental to their active participation and ability to manage their treatment, daily life, and recovery [1–4]. Supporting patient participation and self-management has been widely acknowledged for its positive impact on recovery, overall well-being, and safety [5, 6]. With the rising emphasis on self-management, patients are expected to navigate their care independently, especially evident in the context of cancer, where patients often find themselves managing their disease specific needs beyond clinical settings [5]. It is important for healthcare professionals to acknowledge the patient’s situation, such as their view of and knowledge about the actual illness and the context in which the patient must deal with new circumstances in their life. Lack of understanding and ability to cope with and adapt to changes can cause suffering comprising anxiety, not feeling well, not asking for help and thus lead to delayed recovery as well as complications [5]. Person-centred healthcare has been highlighted as significant, and this concept involves a well-informed patient. However, despite the growing focus on striving to put the patient at the centre, studies show that much more must be done to support patients’ possibilities to participate in their own care [7, 8].

Patients navigating a cancer diagnosis need timely and clear information, but their ability to process it is affected by anxiety, timing, and individual learning capacities. While patients value early information, overwhelming them can reduce recall. Additionally, anxiety negatively impacts information retention, creating a cycle where unmet information needs contribute to distress, which in turn hinders understanding [9–11]. Efforts to enhance patients’ understanding often involve giving them more information, yet studies reveal that this approach can overwhelm patients, leading to confusion rather than clarity [1, 2]. We argue that patients’ struggles to understand and develop the skills needed to cope, self-manage their care, and participate in goal setting for planned treatment should be recognized as a learning process.

In this study we lean on a theoretical pedagogical framework based on a constructivist paradigm. Within the constructivist paradigm the learner, in this case the patient, is viewed as an active participant in processing information cognitively, emotionally, and through practice to construct their understanding individually and in interaction with others and the environment [12, 13]. Prior knowledge, understanding and expectations influence what new or modified knowledge an individual will construct from new learning experiences [12, 14]. Important motivational factors for learning include the will to understand and manage, a sense of meaningfulness, positive experiences of ability, and trust in the current situation [12, 13, 15]. Thus, there is a pressing need to shift from merely providing information to understanding and supporting patients’ learning processes.

To emphasize considerations of learning, we will use the concept pedagogical encounter when referring to situations involving communication with patients about their understanding and coping, that is their learning. The rational for pedagogical encounters rest on the constructivist paradigm described above and involves an encounter, a learning situation, in which all actors interact and learn [16, 17]. This means that healthcare professionals as actors in a pedagogical encounter need to think about how patients learn and how to act to support the patients’ learning.

Significant features of patients’ learning during a serious illness include strong personal motivations to understand and manage the uncertainty, as well as their vulnerability, which challenges their comprehension [1, 2]. Patients need time to emotionally and cognitively process complex information through dialogue with healthcare professionals, and their understanding should be assessed [18]. Pedagogical competence in healthcare teams has long been emphasised as essential in patient care [19]. It is assumed to support patients to process information over time, to manage their new circumstances, and to actively engage in their treatment and care [1, 2].

Nurses play a crucial role in helping patients to develop necessary skills for effective self-management [19]. A scoping review about patients supportive care needs, showed a lack of studies concerning practical support, including information and education [20]. The communication process involved in supporting patients understanding and participation is complex, and healthcare professionals, including nurses, often lack the necessary knowledge in this area [2, 3, 7, 21]. To support patients effectively, health professionals need to understand how people learn, especially when they are vulnerable and ill. This might require time, as newly registered nurses have reported needing more clinical experience to consider pedagogical approaches [19]. To address these challenges, several learning activities focusing on pedagogical skills and knowledge, including the concept of pedagogical encounters, were implemented in a specialist nursing program for cancer care [16]. The activities aimed at empowering experienced nurses to create effective pedagogical encounters that build on knowledge of how patients learn. The learning activities were evaluated by questionnaires and assignments and demonstrated that the students had an increased but varying ability to perform a pedagogical encounter that supports patients’ learning. However, to fully understand how nurses think, act, and what they emphasize in pedagogical encounters, it is essential to explore their stories. Currently, there is a knowledge gap concerning how nurses think and act to facilitate patient learning, a critical factor for patient participation in their treatment and care.

## Methods

### Aim

The aim of this study was to explore experienced nurses’ understanding of the meaning of pedagogical encounters with patients in cancer care, as well as how they think and act during these encounters to support patients’ learning.

### Design

This study uses an exploratory, qualitative interpretative design, within a constructivist paradigm, as it seeks to explore and interpret participants’ experiences, meanings, and perspectives rather than assume an objective reality. This means that the design is in line with the pedagogical theoretical framework. The constructivist paradigm acknowledges that knowledge is co-constructed, shaped by context and prior experiences [12, 13, 22]. To analyse the data, reflexive thematic analysis was employed, emphasizing the researcher’s active role in shaping the analytical process. This approach recognizes that themes are actively generated rather than passively discovered and acknowledges the existence of multiple interpretations [22]. This aligns with the study’s interpretative design, where the goal is to understand and construct nuanced insights into participants’ experiences rather than seek objective categorization.

### Setting

The study was carried out in a specialist nursing programme in cancer care at a university in Sweden. The program focuses on oncology nursing at an advanced level where the students must demonstrate the ability to observe, assess, and address complex care needs in patients requiring cancer care. The program corresponds to 60 credit points over four semesters. On-site training where student apply and integrate theoretical knowledge with practical skills is part of the programme. Further, the programme is offered at half speed, allowing students to combine their studies with clinical work as nurses. Learning activities concerning pedagogical knowledge and skills, including the concept of pedagogical encounters were implemented over three semesters. These learning activities have been described in detail elsewhere [16]. In short, the pedagogical learning activities involved a progressive, reflective learning approach based on constructivist learning theories. Initially, the nurses studied learning theories, research about patient learning and participation and the concept of pedagogical encounter [1, 2, 17, 23]. Then, they observed pedagogical encounters, to apply their insights to clinical practice in cancer care. Further, they planned and conducted pedagogical encounters with patients, receiving and providing peer feedback to enhance their understanding and analysed it in relation to learning theories in a written assignment. Lastly, the nurses led interprofessional seminars to teach healthcare professionals about patient learning.

### Participants

Guided by the model for information power presented by Malterud and colleagues [24] with reflection on the study aim, the specificity of participants in the program, the support by established theory, the ability of the interviews to create strong dialogue, and the study design, not all available participants were invited to the study. Instead, the focus where to ensure variation and richness in data. Therefore, 16 of the 28 nurses enrolled in the specialist nursing program in cancer care were invited to participate in the study. Purposeful sampling was conducted based on age, nursing experience, and diversity in pedagogical knowledge, as demonstrated in a written assignment on pedagogical encounters. Of the 16 invited, eight consented to participate. All participants were women, their median age were 40 years (range 27–51 years), median work experience as nurses were 9 years (range 5–23 years), and their performance on the written assignment was very good (*n* = 4) or good (*n* = 4). Students with poor performance declined to participate in the study.

### Data collection

Data collection was performed through individual interviews using a semi-structured interview guide (Table 1), informed by the research questions and constructed by two of the authors (LEB, CS). Probing questions were used to capture the meaning of their statements, how they act in the encounter and how they think about patients’ learning. Additionally, the students’ written assignments about pedagogical encounters were used as a foundation for the dialogue and the probing questions. The interviews were made at the end of the nurses’ specialist training after they had experience with the pedagogical learning activities and after the assessment and grading of their assignments. The interviews were held either at the nurses’ workplaces at a hospital (*n* = 2), at the university (*n* = 4), or by telephone (*n* = 2), according to the nurses’ choice. The interviews were performed by two of the authors who were also teachers in the pedagogical learning activities (LEB, CS), they lasted between 50 and 70 min, were audio recorded and transcribed verbatim by one person not involved in the research project. The transcripts were checked for accuracy against the audio recordings by one of the authors (LEB) and minor corrections were made.

**Table 1:** The semi-structured interview guide

Questions
If you were to explain the meaning of pedagogical encounters with patients in cancer care to a colleague, what would you say? What would stand out as important?
What do you need to know as a nurse to conduct an optimal pedagogical encounter?
Is there anything you see different when it comes to patients’ learning after this course?

### Data analysis

Data were analysed using the six phases of reflexive thematic analysis [22]. First, three of the authors (TG, LEB, LMP) read the interviews to become familiar with the data and made initial notes of ideas for coding. Then, the whole text was read through again and data in line with the study aim, answering how the nurses think and act to support patient learning, were marked and coded. A code was to describe the meaning of the marked data and could consist of a few words or whole sentences. After that, the codes were transferred into a separate file that served as a coding map. Then, codes in close connection to each other were clustered. The clustering was conducted through an ongoing interpretative engagement and discussion amongst the authors. A preliminary thematic map was drawn by two authors (TG, LMP). The preliminary map was discussed and revised to a new map by the entire group of authors. After that the interpretation of findings and thematic structure were discussed and refined by all authors until names of one overarching theme and five sub-themes were decided upon. Lastly, the final analysis and writing of the findings were led by the first author, who was not involved in the pedagogical learning activities and the data collection, in close discussions with all authors. The analysis was performed using Microsoft Word and Excel and mapping through pen and paper.

To establish rigor of the analysis, the 15-point checklist of criteria by Braun and Clark for good reflexive thematic analysis was followed [22]. All authors are experienced teachers and researchers in nursing and/or medical education. Three of the authors served as teachers for the specific pedagogical learning activities and one worked as a nurse in cancer care and was not involved in the course. Different perspectives and reflexivity were obtained by critical reflection on the researchers’ pre-understanding and its impact on the methodology and interpretation of data. Further, trustworthiness was considered by credibility, transferability, dependability, and confirmability [25]. Credibility is strengthened by the researchers’ diverse yet complementary backgrounds in pedagogy and oncology nursing, offering both clinical insight and understanding of learning processes. To enhance transferability, descriptions of the pedagogical learning activities and participants are provided. Dependability was achieved by following a semi-structured interview guide and starting data analysis after all interviews were completed involving researchers not involved in the data collection. Confirmability was strengthened through regular meetings among researchers during data analysis, revisiting interviews as needed to discuss interpretations. Individual quotations will be used to illustrate the findings.

## Results

**Fig. 1:**
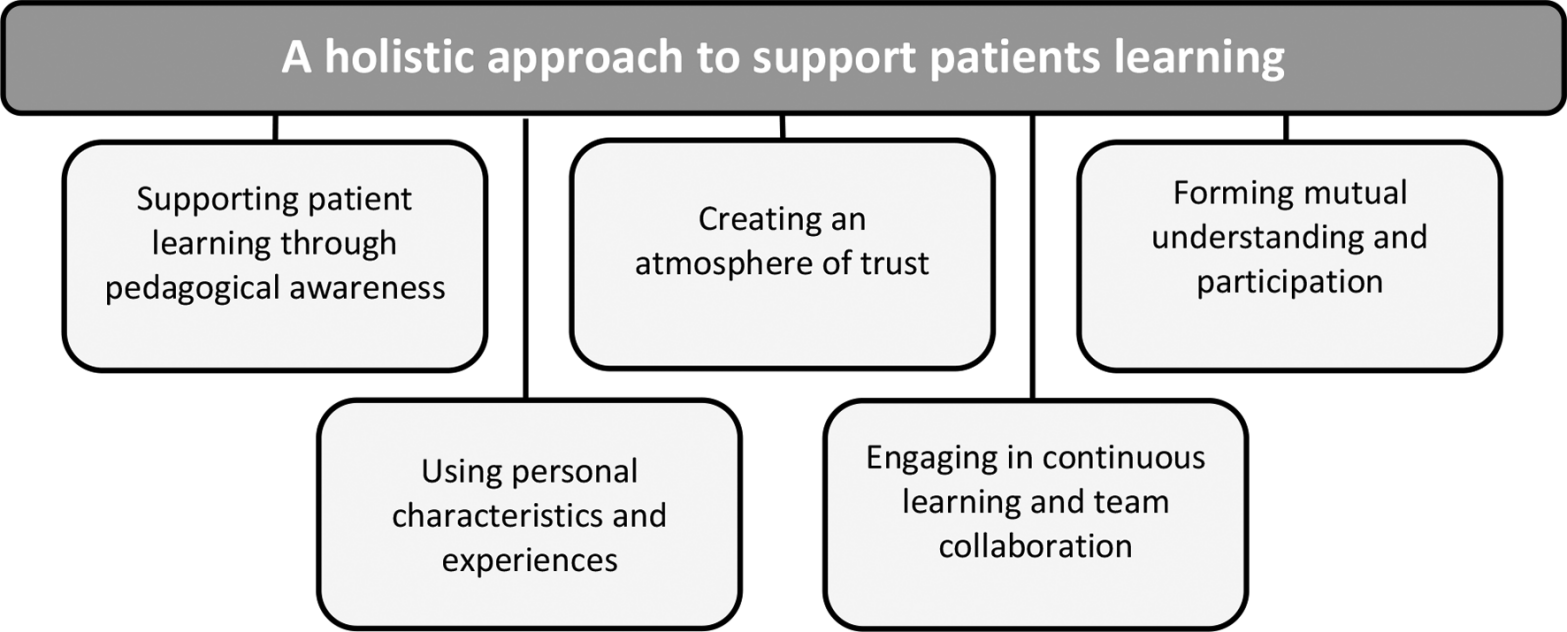
The overarching theme (dark gray rectangle) and the five sub-themes (light gray rectangle) illustrating the experienced nurses (*n* = 8) understanding of the meaning of pedagogical encounters and how they think and act learning during these encounters to support patients’ learning

The nurses’ understanding of the meaning of pedagogical encounters with patients in cancer care and how they think and act in these encounters could be described by one overarching theme “A holistic approach to support patients learning” and by five sub-themes: “Supporting patient learning through pedagogical awareness”, “Creating an atmosphere of trust”, “Forming mutual understanding and participation”, “Using personal characteristics and experiences”, and “Engaging in continuous learning and team collaboration” (Fig. 1).

### A holistic approach to support patients learning

The nurses’ approach to support patients understanding and managing of their illness and care was characterised by recognising the significance of the actual situation as well as the perspectives of the patient, the nurse and the health care team. The implications of this holistic approach and how the nurses think and act related to patients’ learning are further elucidated in the following sub-teams.

### Supporting patient learning through pedagogical awareness

Pedagogical encounters were described by the nurses as diverse, ranging from easy to challenging. The encounters could cover topics like medications, treatments, and coping mechanisms. They could follow a structured plan, but they could also be spontaneous. Pedagogical encounters were explained by the nurses as involving interactive dialogues, emphasizing understanding rather than mere information dissemination. They highlighted the significance of understanding a patient’s background for effective communication. The nurse’s own preparation was described to be key by considering patients’ medical histories and specific topics for discussion. Thereby, accessible materials could be prepared. Likewise, the nurses encouraged patients to prepare for an encounter by noting questions in advance. The nurses found challenges due to time constraints, which hindered in-depth explanations during an encounter. The process of pedagogical encounters was explained not to end with the encounter; rather, continuous learning unfolded. Likewise, follow-ups were essential but varying electronic medical records and systems complicated continuity.

*“I tried to prepare myself*,* but there wasn’t enough information in the medical record I had access to*,* which made the information I had to work with somewhat lacking. I should have found out that the patient was in such a palliative stage as he actually was. But in some way*,* you just must compose yourself*,* try to help as best as you can with what you have at hand*,* and then I could try to make up for what I wasn’t able to prepare for at the time.”* – Nurse 5.

The nurses explained that they had learned that pedagogical encounters demand a toolbox of skills and flexibility to adapt to various patient interactions. The specialist training had prompted reflection on different ways to learn, fostering an environment where individual patient experiences are valued. Likewise, to consider patients prior knowledge and experiences was expressed to be crucial to avoid providing unnecessary information. Some nurses highlighted that, despite not consciously employing a particular theory, they found the specialist training had enhanced their awareness of diverse learning methods and the importance of tailoring nursing interactions to individual needs. The theoretical concepts explored in the specialist training, such as motivation and preunderstanding, was said to have reshaped perceptions of learning, promoting increased awareness. Further, they described how the pedagogical learning activities during their specialist training had broadened their understanding of learning, emphasising finding educational opportunities in unconventional situations. The nurse’s shift in thinking about learning contributed to a change in behaviour during the encounter with the patient.

*“I had a patient who was going to receive a blood transfusion for the first time. I hadn’t thought about it before*,* or well*,* I had thought*,* ‘Now I’ll inform about it*,*’ but now I thought*,* ‘Now she needs to learn what it means for her to receive it.’ I tried more to have a dialogue.”* – Nurse 1.

During the specialist training the significance of person-centred care had become more evident, encouraging the nurses to prioritise patient questions and needs. Additionally, concepts like health literacy had gained prominence, highlighting the challenge patients might face in comprehending medical information.

### Creating an atmosphere of trust

Effective pedagogical encounters were described as characterized by openness, adaptability, and creating a safe space for patients. Meeting in comfortable environments, like home visits, was described to facilitate open discussions. The nurses stressed the importance of being fully present, demonstrating genuine concern for the patient’s well-being.

*“*The pedagogical encounter requires presence—that one is truly there. Not just physically, but also mentally present, listening, trying to read between the lines, or hear between the lines, and asking if something is unclear. To be present, so to speak, with one’s whole being in some way.*”* – Nurse 4.

Furthermore, nurses emphasized the importance of time management, ensuring every interaction was meaningful, regardless of time constraints. The nurses stressed the importance of adaptability and intuition in establishing connections, using techniques like natural pauses, open-ended questions, and a non-judgmental tone to encourage patients to gradually open up. This approach facilitated meaningful conversations, ensuring that patients feel heard, understood, and respected, creating an atmosphere of trust.

*“You can´t tell a person with a drinking problem that needs to quit; ‘hey*,* I heard you’ve been drinking and have a problem with alcohol intake’*,* but I can say*,* ‘here are some advice that I think you should follow in order for the treatment to be as good as possible and that you should be kind to your body*,* we recommend that you eat this and that and avoid alcohol to protect your liver*,*…does that feel okay?’.” – Nurse 8*.

The nurses highlighted the need for humility, flexibility, and active listening during pedagogical encounters. Maintaining eye contact throughout conversations was mentioned as a reliable way to confirm engagement. Further, by giving patients feedback and using subtle probing the nurses could allow patients to share thoughts and fears at their own pace, nurturing trust.

### Forming mutual understanding and participation

The nurses recognized that truly understanding patients’ perspectives requires being mentally present and remaining attentive. To enhance learning, the nurses checked for patient understanding by asking patients to summarize what they’ve been told or if they had any lingering questions and thereby ensuring active patient participation. Using probing questions and feedback loops, including verbal and non-verbal cues and noticing body language and facial expressions like flinching eyes, were described to be crucial to confirm patient understanding.

“*I pay a lot of attention to the eyes. If a person starts to look around or isn’t really focusing*,* um… also their body language a bit… but I think I mostly go by the eyes*,* actually*,* to see where they are at. But also*,* you need to pick up on things and ask because it’s not always easy to notice either. I try to fish for more than just a yes*,* because one can nod even if thinking about something else… and things like that. But also ask: do you understand what I mean here?*” – Nurse 1.

Revisiting topics was important to address queries that could arise later. By asking how patients feel about their treatment or what they understand, the nurses could get an indication of patient satisfaction and address concerns promptly. In situations where patients appear uncertain, the nurses could retain these observations for future encounters, refining their communication techniques.

Understanding and addressing patient reactions were highlighted as crucial components of an effective encounter, as stressed or rushed behaviours can signal confusion or frustration. The nurse emphasized the importance of ensuring clarity and adapting their approach when a patient appeared overwhelmed. Actively involving both patients and their families in the pedagogical encounter was also considered essential, as this collaborative engagement offered diverse perspectives and bridged gaps in patient knowledge. Nurses embraced collaborative learning, recognising that patients often contribute valuable insights from their own investigations and experiences. Moreover, recognizing and addressing the patient’s agenda was seen as vital, ensuring that all questions and concerns are thoroughly addressed, even within time constraints. This process was described as easier when a relationship could be built over multiple encounters, fostering trust and mutual understanding.

*“In cancer care*,* you often have a connection with this person*,* this patient; there’s already an established relationship. As a contact nurse* [nurse with designated responsibility], *I’ve already initiated a process with this person. Then*,* we mutually agree to meet and decide on a time and place*,* having an agenda that both parties agree on”* – Nurse 2.

### Using personal characteristics and experiences

The nurses reasoned that for a pedagogical encounter to be effective, the nurses’ must use their personality, curiosity, and understanding of human nature, often outweighing professional experience and theoretical knowledge. Self-awareness was explained to play a crucial role as nurses must reflect on their own biases and emotional responses when facing challenging situations. A pedagogical encounter could also serve as self-discovery moments, revealing hidden fears or discomfort, such as discussing death. It was believed that a nurse’s insights are enriched by personal experiences, enabling them to empathetically connect with patients. Further, the nurses raised that effective communication demands not only theoretical understanding but also the ability to read individual cues, invite openness, and gently lure meaningful conversations from patients. This was seen as a delicate art of communication, rooted in human insight and humility and believed to be as vital as clinical knowledge in creating impactful pedagogical interactions.

*“I believe that*,* well*,* much can indeed be learned through reading*,* but you also have to experience it. It’s about understanding people*,* you may not be able to talk openly with everyone. You must sense it*,* and if you’re invited*,* then you can talk. But some people don’t want to talk that much*,* and then you must try to extract that little sentence*,* and you can lure it out*,* but that’s also an art. It’s not something you can just learn from reading.”* – Nurse 8.

The nurses raised that a nurse’s confidence in a pedagogical encounter is strengthened by familiarity with the situation. However, this confidence should be balanced with sensitivity and a thorough understanding of the patient’s unique needs. Sorting through a patient’s concerns and aligning them with the essential aspects of their care requires skill and expertise. The nurses believed that the depth of a nurse’s knowledge, especially concerning specific diseases, symptoms, and side effects, significantly impacted their ability to provide effective feedback and support.

*“When you have met enough patients and encountered many similar questions*,* if you are well-read and knowledgeable about lung cancer*,* treatments*,* symptoms*,* potential side effects*,* symptom relief*,* and so on*,* you can draw from your knowledge bank. You use what you know*,* what you understand*,* and what you are familiar with*,* applying it effectively in the pedagogical encounter.”* – Nurse 7.

It was explained that experience brings nuanced insights and a comprehensive understanding of various patient reactions, allowing nurses to tailor their approach. The nurses felt that, while textbooks offer theoretical knowledge, the real art of nursing lies in the ability to navigate diverse patient interactions, adapting strategies based on individual experiences and continuous learning.

### Engaging in continuous learning and team collaboration

The nurses emphasised continuous learning and collaboration. They actively sought information, consulting colleagues of different professions to deepen their knowledge and address patients’ inquiries effectively.

The nurses ensured that patients comprehend medical information by clarifying complex terms and details. Collaboration with physicians was described as crucial and nurses normally attended medical appointments to bridging communication gaps and providing a supportive environment for patients. The nurses described how they often act as a link between physicians and patients, ensuring essential details are not lost. Moreover, the nurses described how they facilitate post-doctor appointment discussions to extracting patients’ unspoken concerns. This was explained to be valuable as their role extends beyond mere medical knowledge, making them offer emotional support and bridge the language of medicine.

“*In medical consultations*,* I think it’s good if the nurse can be present. I find it beneficial to know what the physician has said so that you can complement or refer to that conversation*,* including what was discussed and the information received. In this way*,* it can be useful to recap and know what has already been said*.” - Nurse 5.

Additionally, they strategized within teams to organize comprehensive follow-up plans and address potential concerns. The nurses recognised the significance of a team approach and utilised their expertise. Still, they emphasised their respected boundaries like when questions exceed their knowledge, they consult specialists, fostering a collective effort to enhance patient understanding.

“When I can’t answer all the questions and the patient really want an immediate answer, I usually go and ask the doctor, making use of multiple resources.” – Nurse 3.

## Discussion

The findings from this study provide valuable insights into how experienced nurses think and act in pedagogical encounters with patients in cancer care. They emphasize how well-prepared and thoughtful pedagogical encounters can support patients’ understanding of their treatment, daily life, and recovery. Overall, they demonstrate a strong awareness of the importance of a holistic, person-centred approach to patients’ learning. This resonates with the previous study that followed up the nursing students’ learning in the specialist programme [16]. Their descriptions align with the idea that professionals need to think about how patients learn and how to act to support the patients’ learning, which was evident as they adapt strategies based on individual patient needs, fostering a learning environment [16].

The nurses highlighted the importance of creating meaningful dialogues to build trust and mutual understanding. Further, they demonstrated an awareness of each patient’s individuality, recognizing that different people require different approaches to learning. They stated they had learned this in the specialist programme and their reasoning aligns well with the program’s presentation of constructivist learning theories, which emphasize starting from the individual. Further, this approach has been found to be a prerequisite for facilitating patients’ participation in their care [1]. In a study exploring the meaning of concepts and actions to enhance patients to take part in their care found that establishment of a mutual partnership between a patient and a nurse was essential [26]. The importance of listening and empathy in building trust and supporting patient participation has also been highlighted in other studies [18].

The nurses frequently employed practical techniques such as observing body language and facial expressions to get feedback on the patient’s understanding. This interaction process has been described earlier as a way for nurses to check whether patients’ comprehension is visible or concealed [2]. Further, this is in line with Mezirow’s theory that individuals construct understanding through interactions with others and their environment, leading to a transformed understanding [13]. Additionally, the nurses’ transformed understanding was evident as they broadened their understanding of learning, finding educational opportunities in unconventional situations.

The previous evaluation of the learning activities in the specialist program demonstrated that participants described pedagogical encounters as including the aspects of before, during and after an encounter with a patient [16]. The present study further elucidates that understanding, showing how nurses prepared themselves and encouraged patients to prepare questions before the encounter for a meaningful conversation. This shows that the nurses act in line with a necessary shift in medical oncology education where patient autonomy and collaborative decision-making has been highlighted [27].

The nurses followed up patients’ understanding by asking, observing and interpretating what they saw and heard. However, there was less emphasis on structured follow-up to assess patient learning, except in cases involving contact nurses. Continuity in the nurse’s contact with patients was considered essential to be able to follow-up patients’ understanding. This implies that patients’ learning process and understanding may be difficult to support in the context of short hospital stays, time constraints and when continuity is lacking. Here, more research is needed to be able to educate and support nurses and other healthcare professionals. The role of a coordinating contact nurse may fill that gap as the role has been shown to improve patient-reported outcomes and participation to some extent [28].

The nurses clearly described how they used dialogue, feedback, and flexibility during pedagogical encounters. These are important features described in the learning theories studied in the specialist program. However, the theories behind this knowledge were not explicitly referred to by the nurses. Despite having received theoretical knowledge within pedagogy, the nurses rarely mentioned concepts such as motivation, pre-understanding, meaningfulness, learning processes, or metacognition. Instead, they focused on practical descriptions and their own personal qualities and experiences. This is interesting as they acknowledged that these concepts had increased their awareness of learning. It is possible that integrating new concepts into practice and using them in discussions with colleague requires times. As both Mezirow and Illeris emphasize, learning, particularly transformative or professional learning, is a complex, ongoing process that unfolds gradually as new knowledge is adopted, reflected upon, and integrated into practice [12, 13].

Further, the nurses viewed that the real art of nursing lies in the ability to navigate diverse patient interactions, adapting strategies based on individual experiences and continuous learning. This silent knowledge represents the deep, experiential understanding nurses develop through practice, often without explicitly referring to nursing theories or frameworks. Through the interviews it was clear that the nurses’ narratives were characterized by relational caregiving actions, including active listening, being empathetic, engaging with patients, being present, and supporting and involving families, consistent with nursing frameworks [29]. However, these actions were more often framed as natural abilities rather than connected to formal education and theories of nursing. This gap between practice and theory has been identified earlier and the need for pedagogical competence among other clinical professionals has also been emphasized [19]. Greater familiarity with pedagogical language and frameworks could enhance multiprofessional team discussions and collaborative learning, ultimately improving patient outcomes. This prompts the question of whether adopting a shared pedagogical language in healthcare could improve communication and deepen the team’s understanding of learning processes, thereby enhancing patient care. The importance of enhancing collaboration and communication within multiprofessional teams has been previously recognized, as healthcare professionals may not fully understand the scope of practice of their colleagues and often lack awareness of the information other team members provide to patients [30].

Further, an important insight from the study is the nurses’ descriptions of how their personal qualities shaped patient learning. They frequently mentioned their personal attributes, such as empathy, intuition, and the ability to connect with patients, as central to their work. This is in accordance with Ivarsson & Nilsson [19] who found that some of the newly graduated nurses thought that informing and communicating with someone were natural abilities they possessed before going to nursing school. However, some thought it would be beneficial for them to learn more about pedagogy when they were more knowledgeable in the specialty and more experienced. The authors conclude that nurses need continued education in pedagogy when they have more work experience. In the present study, the nurses had previous work experience, and during their specialist training, they learned both about cancer, its treatment and pedagogy. The previous evaluation of the learning activities, in which the nurses in the present study participated in, showed that the nurses in the specialist program appreciated learning about learning theories [16]. However, given the large and diverse nursing workforce, relying on individual personalities rather than shared professional competencies could lead to inconsistencies in patient care. The knowledge and understanding of how people learn could lead to the ability to meet patients individually, ensuring that all nurses, whether experienced or new to the field, can provide high-quality education for their patients. The integration of such pedagogical concepts into team discussions and practice could also help nurses move beyond intuitive or experiential knowledge and develop a more structured, theory-driven approach to patient care and education. This has been highlighted by Ivarsson & Nilsson [19] who found that interprofessional collaboration promoted information and communication skills.

The nurses’ reliance on clinical experience, rather than formal pedagogical frameworks, also brings to light the challenge of supporting newer nurses. Newly registered nurses who had attended a pedagogical course during their undergraduate training found it difficult to apply their knowledge as they lacked clinical experience. They needed to develop their pedagogical competence continuously and wanted time to reflect on practice together with experienced nurses [19]. In the present study all nurses had several years of work experience and developed their own reflective practices, using their intuition and understanding of how patients learn. But for new nurses who have not yet built this silent knowledge, having a theoretical foundation to guide their interactions could be invaluable. Pedagogical theories could serve as scaffolding for newer nurses, helping them to understand the complexities of patient learning and how to facilitate it more effectively, until they gain the necessary experience. Training in pedagogical skills in nursing education but also in health care organizations has been emphasized [19]. This is particularly relevant in environments where time for patient follow-up is limited, and continuity of care may be lacking. Also in such cases, the role of a contact nurse could be essential in providing ongoing support and ensuring that patients’ learning needs are revisited over time. It has been concluded that an establish relationships between contact nurses and patients can improve patients satisfaction and active participation [31]. However, this is an area where more knowledge is needed.

### Strengths and limitations

Despite the small sample size, the information power is considered high due to the narrow aim, the purposeful sampling, the experienced interviewers who created a high quality dialogue producing rich data and the use of established theories which supported the study design and analyse of data. However, not all invited participants consented to participate in the study. This resulted in less variation than desired, especially in terms of performance on the written assignment, which was not as broad as hoped, as those who had performed poorly chose not to participate in the study. This is an important observation and could be considered a limitation of the study. Also, there were no male nurses enrolled in the course, and thus no male participants available for interviews. Therefore, the study lacks male perspectives, which may influence the transferability of the findings.

The involvement of course teachers in the data collection is another point to consider. There is a risk that the participants wanted to perform well in the interviews due to this and therefore adjusted their dialogue accordingly. However, considering that the interviews were conducted after the course and grading, and given the extent of various and deep descriptions covering the study aim, this risk can be considered limited. Instead, it can be seen as a strength that both the informants and interviewers had experience with the subject matter being discussed. This familiarity could enhance the depth of the discussions. However, a potential bias arises from the possibility that both parties might take certain aspects for granted. To diminish this, the study incorporated reflective discussions and involved multiple researchers in the analysis process. This approach helped ensure a more balanced and comprehensive interpretation of the data.

## Conclusion

The nurses in this study showed an awareness of the importance of patients’ understanding of their health condition and the benefits of participating in their own care and treatment. In their descriptions and reasoning about pedagogical encounters with the patients they demonstrated competence corresponding with pedagogical knowledge, skills and approaches that are required in theory and practice of patient learning. The results point towards the conclusion that three different sources underpinned the nurses’ thoughts and actions in their encounters with patients. Firstly, they used what they had experienced and studied about learning in the program. Secondly, they clearly rested on their competencies in nursing. A third source, strongly expressed by the nurses themselves, was that they relied on their own personal qualities and experiences in their encounters with patients. We would argue that all these sources are significant to support patient learning, but we want to particularly forward the importance of including pedagogical knowledge and practice in health care education. Future research should explore how theoretical knowledge in pedagogy can be better integrated into practice and examine how healthcare environments can be structured to support nurses in their pedagogical roles, particularly in time-constrained settings.

## Data Availability

The datasets analysed during the current study are not publicly available since individual privacy could be compromised but are available from the corresponding author on reasonable request.
